# Anaplastic thyroid tumor as an embolic source of metastasis

**DOI:** 10.1016/j.jvscit.2022.05.006

**Published:** 2022-06-30

**Authors:** Paloma González Rodríguez, Sara Mercedes Morales Gisbert, Jose Ignacio Chiriboga Granja, Maria Cano Medina, Francisco Julián Gómez Palonés

**Affiliations:** a Department of Angiology, Vascular and Endovascular Surgery, Hospital Universitario Doctor Peset, Valencia, Spain; b Department of Pathological Anatomy, Hospital Universitario Doctor Peset, Valencia, Spain

**Keywords:** Anaplastic, Cancer, Ischemia, Peripheral arterial disease, Thyroid carcinoma, Vascular surgical procedures, Venous thromboembolism

## Abstract

Tumor-based arterial thromboembolism in patients with cancer is a poorly described concept that lacks evidence for surgical indications owing to its unusual occurrence. The study and understanding of this condition’s etiology is, however, essential because it could constitute the initial presentation or determine the prognosis of oncologic disease. In the present report, we have described the case of a 77-year-old female patient with multiple cerebral, splenic, and upper limb arterial embolic episodes. Embolectomy for acute upper limb ischemia revealed the histopathologic diagnosis of an anaplastic thyroid tumor.

The association between thrombosis and cancer has been widely recognized as venous thromboembolic disease that represents the second leading cause of death of cancer patients.^1^, ^2^, ^3^, ^4^ Although the literature has mainly involved venous thrombosis, cancer-associated arterial ischemic events can occur in 1.5% to 3% of patients.^3^^,^^5^^,^^6^ The mortality of patients with acute limb ischemia (ALI) has been comparable to that of cancer patients with venous thromboembolism (VTE).^7^^,^^8^

The pathogenesis of arterial ischemic events in oncologic patients is complex and greatly influenced by multiple factors.^5^ ALI due to thromboembolism can present as an early symptom of occult cancer. Thus, appropriate etiologic studies must be performed because a delay in the diagnosis can affect the prognosis and survival of cancer patients.^9^ Atrial myxoma has been frequently described as the most common cause of tumor-based arterial thromboembolism.^10^^,^^11^ However, ALI resulting from direct embolism due to a nonmyxomatous tumor is an extremely unusual entity.

## Case report

A 77-year-old woman was admitted to the emergency room with transient paresis of her left arm with full recovery. She was transferred to the neurology department because of the suspicion of a transient ischemic attack. The cardiac examination, carotid Doppler ultrasound, and cranial computed tomography angiography (CTA) did not show any pathologic findings.

**Fig 1:**
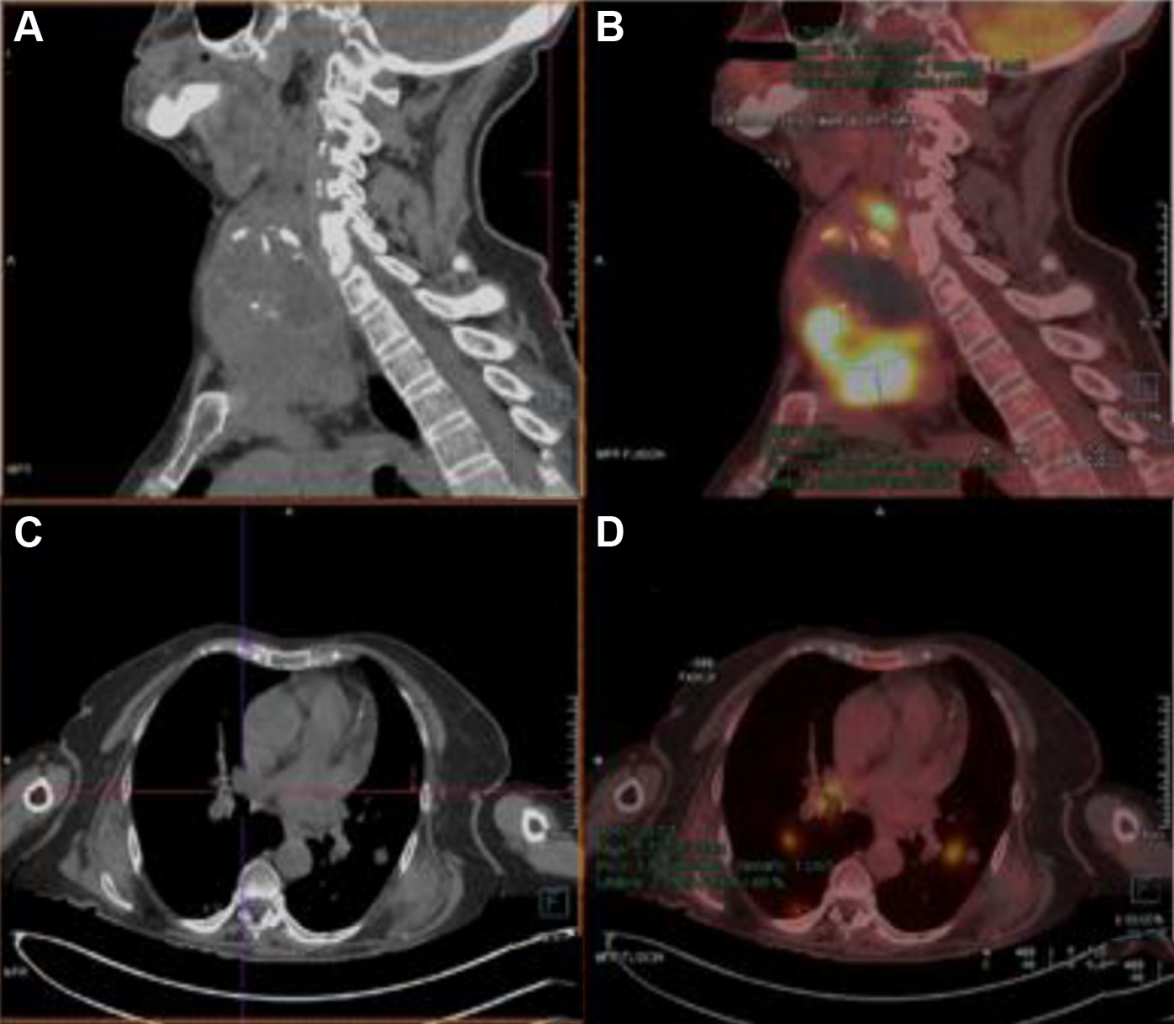
**A, B,** Sagittal computed tomography and positron emission tomography scans showing a multinodular goiter and a hyperintensity focus (tumor). **C, D,** Cross-section computed tomography and positron emission tomography scans showing an intracardiac thrombus and a hyperintensity focus (tumor).

Using thoracoabdominal CTA, an image indicative of a thyroid tumor was incidentally found, with local extension and invasion of the pulmonary vein and left atrium, in association with pulmonary bilateral metastasis. Fine needle aspiration of the thyroid tumor was performed. The findings from magnetic resonance angiography ruled out the presence of intracranial disease. Finally, positron emission tomography was performed (Fig 1).

The patient remained asymptomatic and started anticoagulant treatment. However, a few hours later, she began complaining of sudden severe pain in her upper right limb, with radial nerve palsy. On examination, the radial pulse had been lost, and her hand was cold and painful. Emergency Doppler ultrasound and CTA confirmed ALI due to radial artery occlusion, with imaging findings compatible with splenic infarcts that suggested an embolic origin.

**Fig 2:**
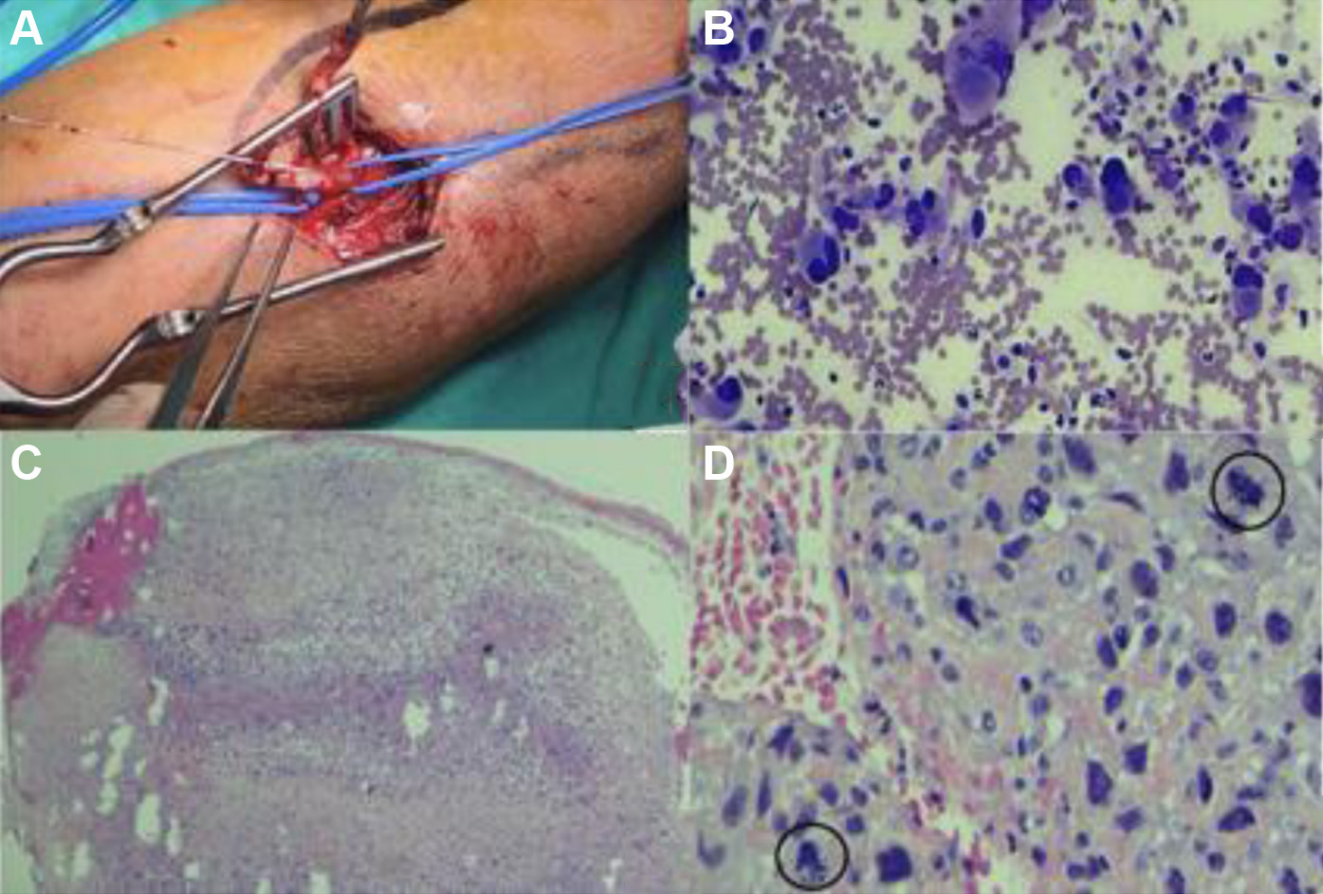
**A,** Intraoperative radial embolectomy. **B,** Fine needle aspiration of the thyroid showing densely cellular extensions with pleomorphic elements, anisonucleosis, broad weakly eosinophilic cytoplasms, and frequent mitotic figures. **C, D,** Organized fibrin-hematic material, including cells with characteristics superimposable on those identified from the fine needle aspiration, distributed in isolation and in three-dimensional clusters of variable size, was identified. **D,** Immunohistochemical study showing co-expression of vimentin and keratins (CAM 5.2) and immunoreactivity for PAX8.

The patient was quickly taken to the operating theater. Radial embolectomy was performed through a proximal radial approach under local anesthetic, and atypical embolic material that was pale-white looking with a fleshy consistency (Fig 2) was found and submitted for histopathologic examination. After surgery, the patient’s radial pulse and neurologic functionality had recovered.

One week later, the histopathologic examination had identified an undifferentiated (anaplastic) thyroid carcinoma (category VI using the Bethesda classification) in both the fine needle aspiration and the embolic clot sample. Further investigation with echocardiography did not show any intracardiac thrombus.

The patient refused chemotherapy. By the first follow-up examination 1 month later, she had not experienced any new embolic event. However, the cancer had spread, with the appearance of brain metastases. At 2 months after the intervention, she had died of bilateral COVID-19 (coronavirus disease 2019) pneumonia. The patient provided written informed consent for the report of her case details and imaging studies.

## Discussion

Vascular pathology acquires a special relevance for cancer patients owing to the greater prevalence compared with that in the general population. Arterial ischemic events (AIEs) have not been widely studied despite their association with a poor prognosis. The mortality from ALI has been comparable to that of VTE in cancer patients (≤11% with pulmonary thromboembolism).^3^^,^^12^

It has been reported that owing to the recent improvements in oncologic, the survival rates have increased but with a greater incidence of AIEs.^2^^,^^5^ Multicenter studies have found that the incidence of ALI will be doubled and the mortality rate tripled in the case of solid tumors in patients with cancer compared those of a control group.^7^ Another important factor in the occurrence of AIEs is the vascular toxicity resulting from chemotherapeutic agents, such as bevacizumab, which will multiply the incidence of AIEs by 1.4, regardless of the dose.^5^^,^^7^

Furthermore, the occurrence of ALI in patients with cancer might be related to less frequent phenomena such as nonbacterial thrombotic endocarditis, paradoxical embolism from VTE, or tumor-based arterial thromboembolism. Arterial embolism can present as a paraneoplastic syndrome in ≤10% of those with cancer.^11^ The tumors with the highest prevalence of AIEs have been lung, digestive, gynecologic, prostate, and hematologic.^6^^,^^12^^,^^13^, ^14^, ^15^, ^16^ The most commonly embolized anatomic regions have been the supra-aortic trunks, limbs (with the incidence in the lower limbs four times greater than that in the upper), and visceral arteries.^6^^,^^15^

The current clinical guidelines for the management of ALI have recommended that “for patients with ALI and underlying malignant disease, active revascularization in selected patients should be considered” (evidence level, class 2A, level B).^9^ Multidisciplinary management is mandatory, especially for patients with cancer. Urgent surgery was indicated for our patient because she had developed grade IIB (immediate threat) ALI in her arm.^9^ No evidence or guidelines are available that have recommended any type of thromboprophylaxis against thromboembolic events in patients with cancer.^12^^,^^13^^,^^17^

Limb loss and mortality have been doubled in patients with a diagnosis of cancer within the 24 months before the episode of ALI compared with those in patients without cancer.^13^ Nevertheless, several studies have shown comparable perioperative survival in the treatment of ALI in patients with cancer compared with the general population,^9^ despite the lower short-term survival (20% vs 50% at 6 months).^11^^,^^18^ Tsang et al^18^ reported an 87.5% survival rate and 63% limb salvage rate, and Mouhayar et al^19^ reported an 80% amputation-free survival rate at 1 year. Although the short-term mortality after surgery has been good,^10^ a bias resulting from the presence of a tumor in an advanced stage that aggravates the prognosis has been reported.^20^^,^^13^

Some studies have shown that the onset of atherothrombotic events will occur in most cases shortly after the cancer has been diagnosed.^12^^,^^16^^,^^19^^,^^21^ Sundbøll et al^13^ identified acute lower limb ischemia due to thrombosis as a marker of occult cancer with a 2.5% risk of cancer at 6 months.^13^^,^^16^

Thromboembolic diseases have been considered markers of occult neoplasms and an indication of a poor prognostic factor for cancer patients.^12^^,^^11^ For patients with arterial embolisms, it is necessary to determine the cause of the embolism and, if possible, treat the cause to avoid new embolic or ischemic events.^10^^,^^13^^,^^14^ Individualized management adjusted to patient-specific conditions can prove effective; however, no consensus has been reached regarding the management of ALI in oncologic patients, for which the documented evidence has been only isolated cases. Screening for an unknown cancer should be performed when no intra-arterial factors or embolic cardiac origin related to an ALI have been found.^11^^,^^17^

## Conclusions

ALI in oncologic patients is an underreported condition despite its high morbidity and mortality. Although ALI might be a poor prognosis marker for patients with cancer, at present, it is not indicated to perform tumor screening for all patients presenting with ALI. However, a complete etiologic study might be effective for patients with an atypical clinical presentation or an unknown cause of ALI. Optimizing the management of ALI in cancer patients will minimize the potential sequel of untreated acute ischemia and, thus, improve the quality of life for oncologic patients despite their limited life expectancy.
